# TCAD Simulation Studies on Ultra-Low-Power Non-Volatile Memory

**DOI:** 10.3390/mi14122207

**Published:** 2023-12-06

**Authors:** Ziming Xu, Jinshun Bi, Mengxin Liu, Yu Zhang, Baihong Chen, Zijian Zhang

**Affiliations:** 1 Institute of Microelectronics of Chinese Academy of Sciences, Beijing 100029, China; xuziming@ime.ac.cn (Z.X.);; 2 University of Chinese Academy of Sciences, Beijing 100049, China; 3 Institute of Microelectronics of Tianjin Binhai New Area, Tianjin 300308, China; 4 Beijing Zhongke New Micro Technology Development Co., Ltd., Beijing 100029, China; 5 Institute of Semiconductors, Chinese Academy of Sciences, Beijing 100085, China; 6 Shanxi Key Laboratory of Advanced Semiconductor Optoelectronic Devices and Integrated Systems, Jincheng 048026, China; 7 Jincheng Research Institute of Opto-Machatronics Industry, Jincheng 048026, China

**Keywords:** emerging memory, non-volatility, low-energy switching, resonant tunneling structure

## Abstract

Ultra-Low-Power Non-Volatile Memory (UltraRAM), as a promising storage device, has attracted wide research attention from the scientific community. Non-volatile data retention in combination with switching at ≤2.6 V is achieved through the use of the extraordinary 2.1 eV conduction band offsets of InAs/AlSb and a triple-barrier resonant tunnelling structure. Along these lines, in this work, the structure, storage mechanism, and improvement strategies of UltraRAM were systematically investigated to enhance storage window clarity and speed performance. First, the basic structure and working principle of UltraRAM were introduced, and its comparative advantages over traditional memory devices were highlighted. Furthermore, through the validation of the band structure and storage mechanism, the superior performance of UltraRAM, including its low operating voltage and excellent non-volatility, was further demonstrated. To address the issue of the small storage window, an improvement strategy was proposed by reducing the thickness of the channel layer to increase the storage window. The feasibility of this strategy was validated by performing a series of simulation-based experiments. From our analysis, a significant 80% increase in the storage window after thinning the channel layer was demonstrated, providing an important foundation for enhancing the performance of UltraRAM. Additionally, the data storage capability of this strategy was examined under the application of short pulse widths, and a data storage operation with a 10 ns pulse width was successfully achieved. In conclusion, valuable insights into the application of UltraRAM in the field of non-volatile storage were provided. Our work paves the way for further optimizing the memory performance and expanding the functionalities of UltraRAM.

## 1. Introduction

Memory technology plays a crucial role in the field of computing. Currently, mainstream types of electronic memory include DRAM and Flash memory, which complement each other in terms of cache, main memory, and data storage. However, these types of memory still have certain limitations that restrict their performance in practical applications. On one hand, DRAM can deliver fast operating speeds but faces several issues, such as data loss [1] and the need for periodic refreshing. On the other hand, Flash memory, which utilizes floating gates (FGs) to achieve non-volatility, introduces additional costs. In addition, there is a possibility of voltage-induced failure mechanisms within the oxide layer during the execution of high-voltage write and erase operations [2], thus limiting the device’s durability. To overcome these challenges, a comprehensive solution is sought by the scientific community which is known as “universal memory”. This type of memory should possess enhanced characteristics, such as non-volatility, low voltage, low power consumption, non-destructive readout, cost-effectiveness, ultra-high speed, and high durability. The fabrication of such a type of memory would be applicable to various storage requirements that could lead to the development of the computing paradigm. However, a seemingly contradictory requirement between non-volatile and low-power write and erase operations exists, raising questions about the feasibility of this universal memory.

In the quest for a more comprehensive solution [3], various emerging memory technologies have been explored [3] which are collectively known as “storage-class memory” (SCM) [4] and include phase-change memory [5,6], ferroelectric RAM, resistive RAM [7], conductive-bridge RAM [8], and magnetoresistive RAM [9]. These emerging memory technologies have shown promising results and arise as potential solutions for achieving universal memory.

In recent years, Ultra-Low-Power Non-Volatile Memory (UltraRAM), as an emerging memory technology, has attracted considerable attention from both the scientific community and industry and demonstrated significant potential in the development of novel memory devices [3]. UltraRAM adopts a Flash memory structure composed of InAs/AlSb/GaSb heterostructures and achieves non-volatility through the integration of floating gates and double quantum well structures. Although this type of configuration offers many comparative advantages, such as low power consumption [10], high density, and long-term storage retention [11], currently, UltraRAM still faces various challenges including a limited storage window size, threshold voltage drift, and switching speed limitations. To effectively overcome these issues, various improvement strategies have been proposed to enhance the performance of UltraRAM [12].

In this direction, in this work, the storage principles and device performance of UltraRAM were extensively investigated by conducting TCAD software (Crosslight PICS3D version) simulations. Based on electrical test results, an improvement strategy was proposed by reducing the thickness of the channel layer to increase the storage window size and optimize device performance. More specifically, devices with 60 nm, 40 nm, and 20 nm channel layers were simulated, and their charge distribution and performance were analyzed. The simulated results validated the feasibility of the improvement strategy and demonstrated that thinning the channel layer can significantly increase the storage window and improve the threshold voltage characteristics of a device. Additionally, the switching speed of each device was thoroughly examined by conducting tests on the floating gate charge and transfer characteristics with the application of different pulse durations and analyzing the device’s switching speed. Although the current speed still falls short of the ideal speed of UltraRAM, this work provides valuable insights for further improving the performance of UltraRAM.

## 2. Study of Device Structure and Operating Mechanism

**Figure 1:**
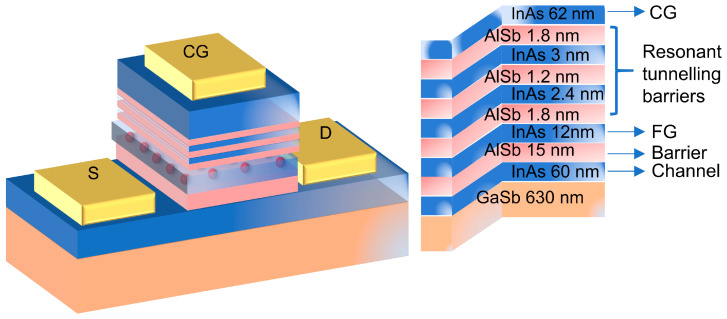
Device structure: a schematic illustration of the processed device with control gate (CG), source (S), and drain (D) contacts (gold). The red spheres represent stored charge in the floating gate (FG).

UL TRARAM™ is a novel non-volatile logic memory utilizing the quantum mechanical resonance tunneling effect, aimed at achieving high performance with low switching energy. It employs InAs and AlSb as materials for the tunneling region, with InAs being a high-mobility material characterized by an extremely low effective mass, making it suitable for applications requiring the confinement of energy in high quantum well (QW) structures. AlSb provides a 2.1 eV barrier that creates a band offset with InAs. These III-V group materials have similar lattice constants, enabling high-quality layer growth. This work utilized the Crosslight TCAD software package for performing simulation studies and constructed the principal structure of UltraRAM, as can be observed in Figure 1, which resembles Flash memory but lacks an oxide layer as a barrier. Instead, the conduction band energy alignment principle in the 6.1 Å semiconductor series [13] was utilized, and an InAs/AlSb resonant tunneling (RT) structure above the floating gate (FG) was introduced, making the device structure beneath the storage unit more similar to a high-electron-mobility transistor (HEMT). Additionally, to compensate for background doping and natural defects in the underlying GaSb [14], n-type doping was applied to the InAs channel layer at a concentration of 5 × 10^17^ cm^−3^ with Si dopants, while intrinsic materials were used for the RT structure. Additionally, a doping concentration of 10^18^ cm^−3^ with Si dopants was set for the InAs layer placed on the RT structure.

**Figure 2:**
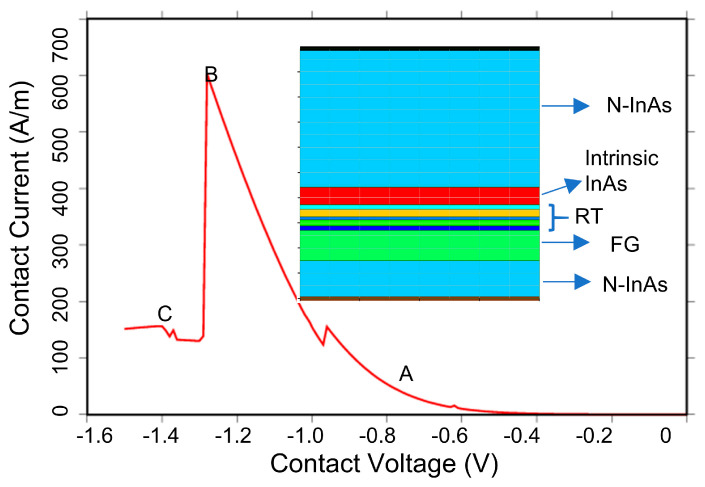
I-V characteristic curve of the diode based on the resonant tunneling structure.

**Figure 3:**
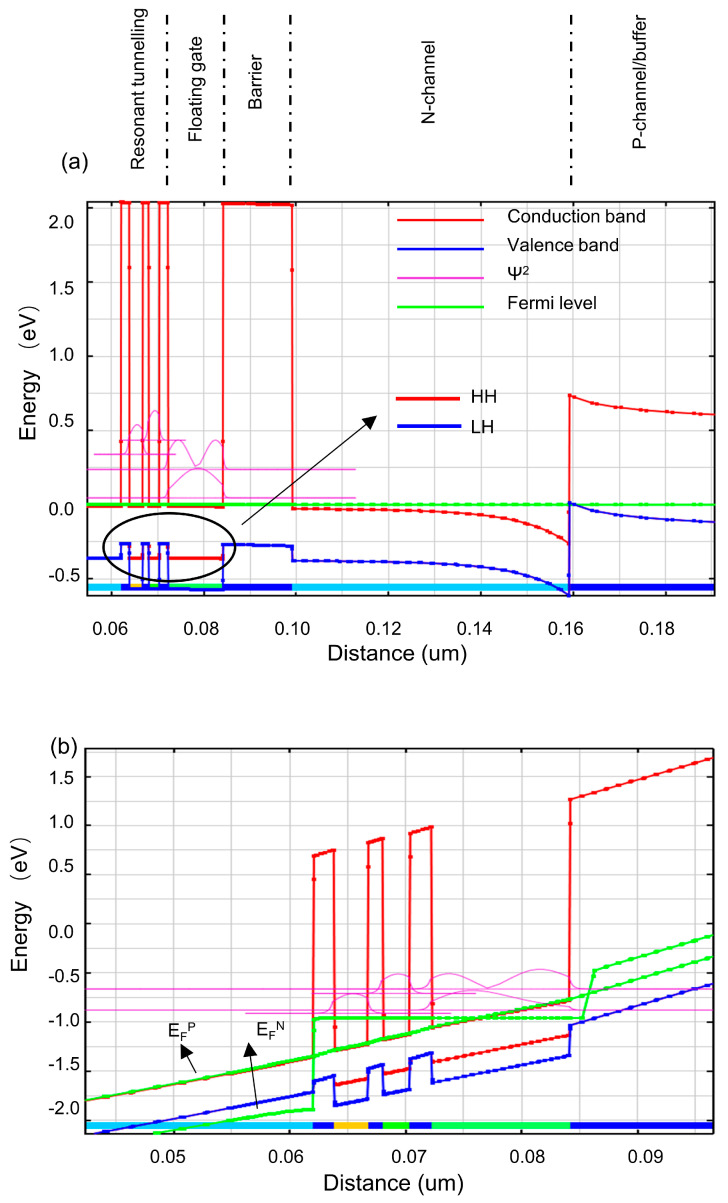

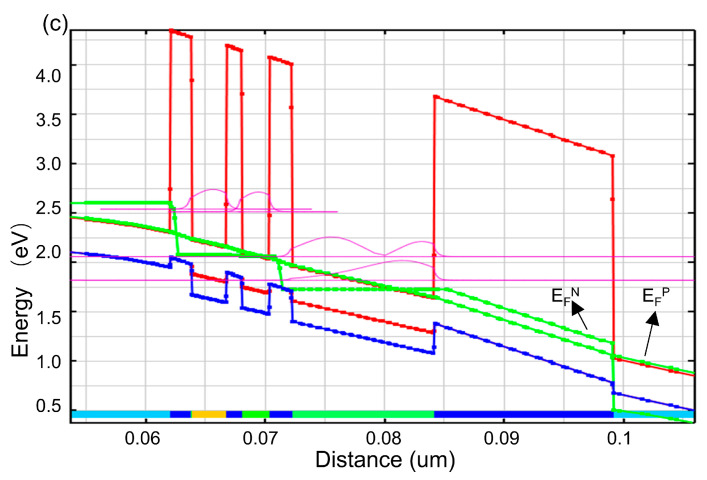
The calculated band diagram plotted vertically through the structure: (**a**) In the absence of bias across the device. (**b**) At the end of the erase process with V_CG-S_ = +2.6 V, showing the depletion of electrons in the FG. (**c**) At the end of the write process when V_CG-S_ = −2.6 V, resulting in a significant density of electrons in the FG.

The achievement of excellent non-volatility properties relies on the double quantum well structure above the floating gate in UltraRAM. Therefore, an initial model of the double quantum well structure (AlSb/InAs/AlSb/InAs/AlSb) in the UltraRAM device was first constructed, and voltage was applied to obtain its I-V characteristic curve (Figure 2). This step aimed to study the resonant tunneling structure, which is the most critical aspect of UltraRAM. The I-V curve reflects the resonant tunneling characteristics of the double quantum well (QW) structure. Based on the analysis of the I-V results combined with the band diagram of the complete device, the working mechanism of the double QW structure can be elucidated. In the low voltage range (labeled as region A-B), the energy bands of the two QWs are not aligned, resulting in a higher lowest energy level in QW1 than in QW2. Therefore, electron tunneling cannot occur at this stage, as shown in the corresponding band diagram in Figure 3a. However, when a certain threshold gate voltage is applied, the energy bands of the double QW structure become aligned and level matched, resulting in the same lowest energy level in both QWs, which enables resonant tunneling to occur. At this point, electrons tunnel through the QW, corresponding to the peak current point (B) on the curve, as shown in the band distribution of the QW in Figure 3b. As the applied voltage increases further, the energy band of QW1 exceeds that of QW2, causing an energy band mismatch and preventing electron resonance tunneling, leading to a rapid drop in current, as seen in region B-C. This simulation result reflects the main features of the double QW structure: when the energy levels of the QWs are mismatched, the structure exhibits a strong blocking ability against electrons, resulting in low current; when the energy levels of the QWs are matched, a lower external voltage is required, and the current rapidly increases. Therefore, it is preliminarily predicted that UltraRAM devices have excellent non-volatile, low-operating-voltage, and low-power-consumption advantages.

After verifying the advantages of the RT structure, a complete model of UltraRAM was developed, and subsequent experiments were conducted. Figure 3a depicts the simulated band alignment at the InAs/GaSb interface. The probability densities, Ψ^2^, for the position of the electrons in the QWs and FG are plotted in arbitrary units. It is worth noting that band splitting occurs in the QW and FG regions, resulting in the presence of two valence bands: a heavy-hole valence band (HH) depicted in red and a light-hole valence band (LH) depicted in blue. By observing that the conduction band of InAs is lower than the valence band of GaSb, it was found that electrons flow from the GaSb to the InAs, while holes are formed in the GaSb. The conductivity of the entire channel is dominated by the electrons in the InAs due to their higher mobility and surface density induced by doping. The complete UltraRAM structure includes an intrinsic InAs floating gate (FG) separated by a 15 nm AlSb barrier layer from the InAs channel and forms a double InAs quantum well with a triple AlSb barrier between the FG and the n-doped InAs control gate (CG), leading to the formation of a resonant tunneling barrier. These quantum wells have different thicknesses and energy-constrained states. In an unbiased system, the manifestation of direct electron tunneling between the FG and CG is blocked, and there is no charge flowing into or out of the FG, achieving non-volatility. However, with the application of a small voltage to the CG, adjustable coupling of the energy levels for write and erase operations can take place. Through simulation tests, it was found that the absolute value of the bias voltage required for erase or write operations is ≤2.6 V.

Figure 3b,c depict the calculated band diagrams during the erase and programming processes when a bias voltage is applied to the CG. A slight difference is that when an external bias is applied, the non-equilibrium state causes the unified Fermi level to split into an electron quasi-Fermi level (E_F_^N^) and a hole quasi-Fermi level (E_F_^P^), as indicated in Figure 3b,c. More specifically, when a + 2.6 V erase voltage is applied, the electron energy level of QW1 in the quantum well structure is lower than that of QW2, both of which are below the first excited state and very close to the ground state level in the FG. Additionally, the calculated FG ground state electron probability density shows a significant electron accumulation at the interface with the resonant tunneling barrier, which is accompanied by a gradually decaying tail extending to the left side of the first AlSb barrier. Thus, the erase operation causes electrons to flow from the FG to the CG, resulting in a reduction in the charge in the FG. In the programming operation, which involves the application of an external bias of −2.6 V, the band bends downwards, and the energy levels of QW1 and QW2 almost overlap. Therefore, a strong coupling and resonant tunneling between these levels is induced, causing the transfer of electrons from the CG to the FG, charging the FG.

The data reading of the UltraRAM device was achieved conveniently by measuring the source-drain current (I_S-D_) at a fixed source-drain voltage (V_S-D_). Due to capacitive coupling between the FG and the channel, the conductivity of the channel depends on the charge stored in the FG. Therefore, data reading was accomplished by measuring the source-drain current. In the programming state, the source-drain current was lower, indicating an increased charge in the FG which decreased the conductivity of the channel. In the erase state, the source-drain current was higher, indicating electrons leaving the floating gate and an increased conductivity of the channel. By comparing the charge distribution before and after the programming procedure, the basic principle of UltraRAM can be more intuitively demonstrated, which involves the modulation of the channel’s conductivity by adjusting the electron density in the floating gate. Before programming, the channel contains a large number of electrons, while after programming, electrons enter the FG, leading to an increase in the electron concentration in the floating gate. As a result, the electron density of the channel layer is reduced, and the conductivity is decreased.

**Figure 4:**
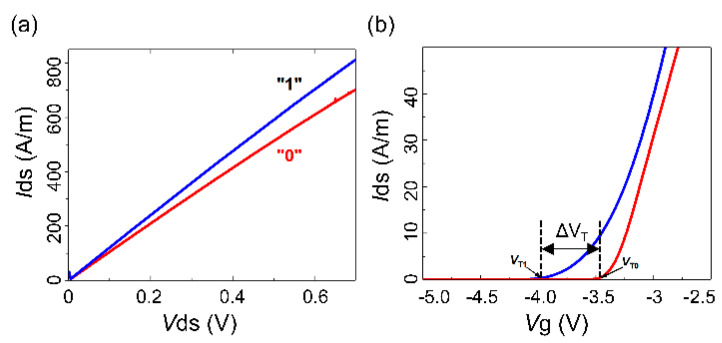
(**a**) Output characteristic curve; (**b**) transfer function curve.

Figure 4 displays the electrical characteristic curves of the UltraRAM device in two different states. The programming state is defined as “0”, where a partial depletion of the electrons occurs in the channel, resulting in a decrease in the source-drain current. The erase state is defined as “1”, where electrons leave the floating gate, and compared to the “1” state, the source-drain current increases. By analyzing the output characteristic curves in Figure 4, it can be observed that both states have a negative bias for the threshold voltage. Considering the analysis of the charge distribution diagram, this phenomenon could be ascribed to the excessively large thickness of the channel layer, which prevents complete electron depletion in the “0” state. Thus, a certain negative bias voltage must be applied to turn off the device. In the “1” state, partial depletion of the electrons occurs in the channel, causing the threshold voltage to shift to the right. Conversely, in the “0” state, the threshold voltage shifts to the left, resulting in a transfer characteristic with a storage window.

## 3. Optimization of the Channel Layer and Analysis of the Pulse Time

### 3.1. Optimization Scheme for a Clearer Memory Window in UltraRAM

So far, the structure and the storage mechanism of UltraRAM have been introduced and its advantages have been validated through its band structure and storage principles, resulting in a storage window of approximately 0.5 V. However, there are still some issues that need to be addressed, such as the lack of clarity in the storage window and whether the actual speed can approach SRAM. These limitations restrict the development of UltraRAM in large-scale array applications. Therefore, based on the mechanism of UltraRAM, an optimization scheme was proposed to improve the clarity of the storage window.

Based on our previous analysis, capacitive coupling between the floating gate charge and the channel is crucial for controlling the storage state of the device. Therefore, it can be argued that by thinning the channel layer, the floating gate can better control the channel, thus addressing the issue of a small storage window. To validate the feasibility of this improvement scheme, three sets of channel layer thicknesses were designed, namely 60 nm, 40 nm, and 20 nm, and further research was conducted.

**Figure 5:**
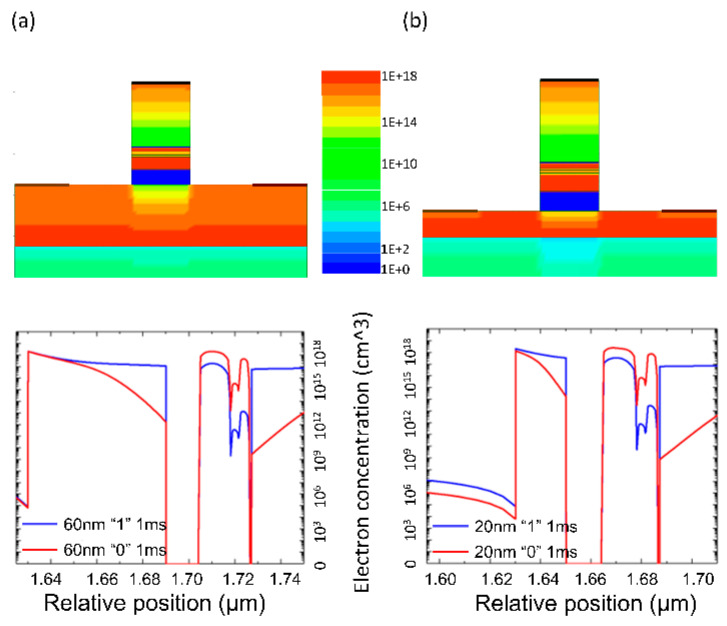
The charge distribution maps after programming for the improved device: (**a**) 60 nm initial device; (**b**) 20 nm improved device.

**Figure 6:**
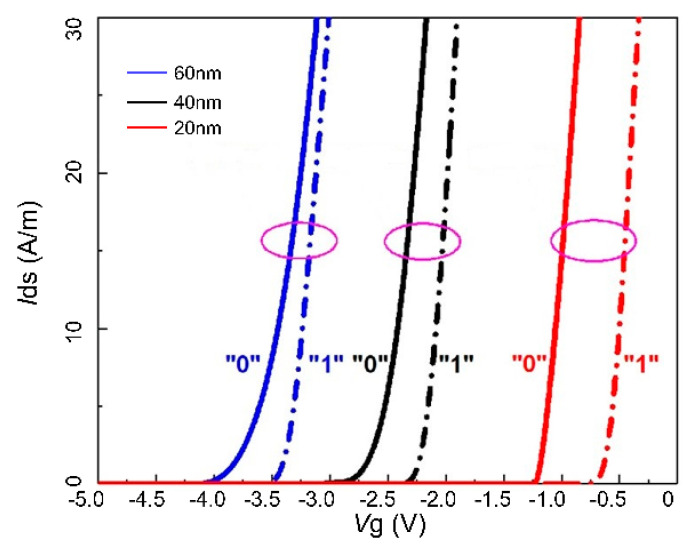
Transfer characteristic curves for devices with different channel layer thicknesses (60 nm, 40 nm, and 20 nm).

Figure 5 illustrates the charge distribution after programming for devices with a 60 nm channel layer and a 20 nm channel layer (the results for the 40 nm channel layer device, which align with expectations, are not shown here). The results confirm the feasibility of the improvement scheme. In the 20 nm channel layer device, the floating gate charge effectively depletes the entire channel layer, showing an overall decrease in the charge compared to the partial depletion in the prototype device. This phenomenon results in changes in device performance, as shown in Figure 6. The storage window of the device significantly increases in the thinned-channel-layer device due to the enhanced control of the floating gate over the channel, resulting in an 80% improvement in the storage window compared to the prototype device, enabling a window of approximately 0.9 V. Furthermore, as the channel layer is thinned, the threshold voltage of the device further shifts to the right, which is consistent with our expectations.

### 3.2. Analysis of Switching Speed for UltraRAM under Different Pulse Widths

Although the simulation results have successfully validated the feasibility of the improvement scheme, we aim for the scheme to optimize the storage window without significantly sacrificing other aspects of device performance. In addition to the previously discussed low operating voltage and long-term storage retention capability, the fast-switching speed of UltraRAM is also regarded as an important characteristic. In the preliminary simulations, relatively long write/erase pulses were used for conservative purposes. Therefore, the switching speed of the device was further investigated, as well as the extent to which the improvement scheme affects the switching speed.

Considering that the state transition of the memory device is achieved through the depletion effect controlled by the floating gate charge, the transfer characteristics and floating gate charge levels were analyzed under the application of different pulse widths to assess the device’s switching speed. Transient simulation results from previous studies [15] demonstrated that the ideal switching speed of UltraRAM should be below 5 ns.

**Figure 7:**
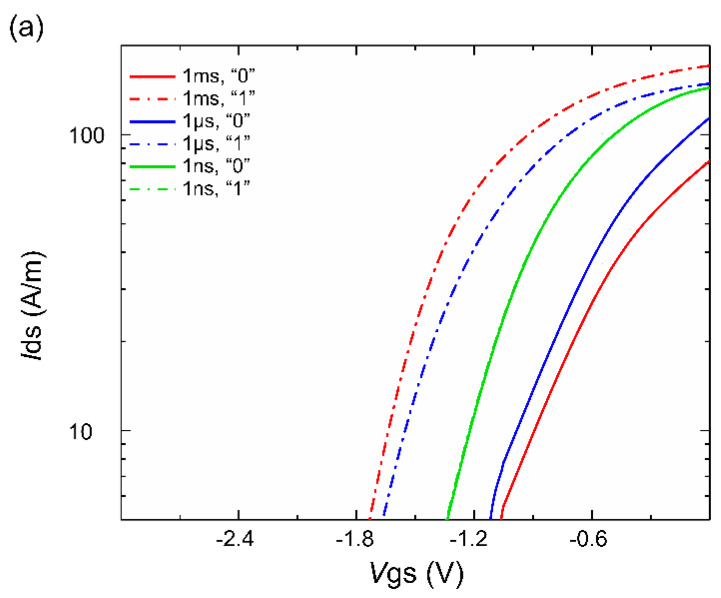

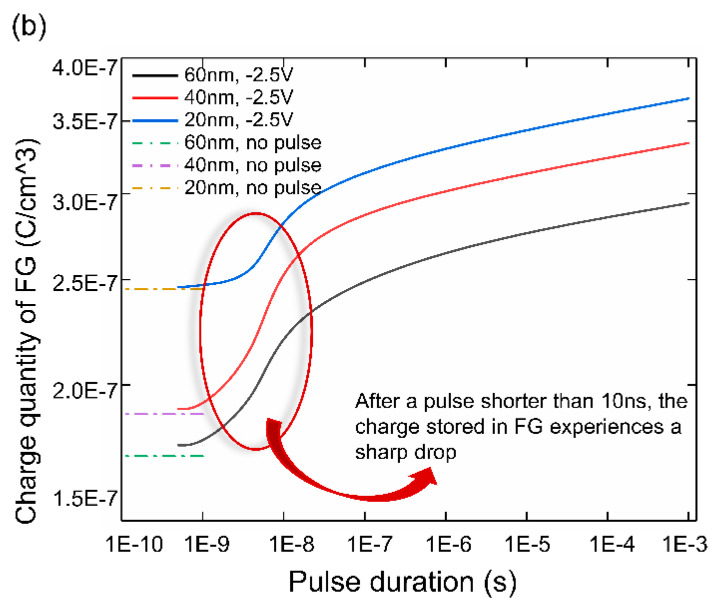
(**a**) The transfer characteristic curves for different pulse durations. (**b**) The relationship between the write pulse duration and the change in the floating gate charge.

Therefore, in this work, pulse widths ranging from 1 ms to 500 ps were tested, and the transfer characteristic curve for the 20 nm channel device is presented in Figure 7a. Pulse widths of 1 ms, 1 μs, and 1 ns were tested. From the results shown in Figure 7a, it can be observed that the device successfully generated a storage window under the application of pulse widths of 1 ms and 1 μs, indicating that effective programming and erasing operations can be achieved with these two pulse widths. However, with a pulse width of 1 ns, the two curves shown in the graph are very close and almost overlap. This suggests that under the latter programming condition, the device cannot perform normal write and erase operations. This may be due to the pulse width being too short to provide sufficient time to complete the programming and erasing processes, resulting in an indistinct storage window. To further investigate the theoretical response speed of the device, devices with different channel layer thicknesses were examined, and several sets of pulses ranging from 1 μs to 1 ns were added to output the floating gate charge levels for analysis. The results are depicted in Figure 7b.

In the work, the floating gate charge levels of UltraRAM with different channel layer thicknesses under different pulse widths were systematically analyzed to study their response speed. The results showed that the channel layer thickness may have some influence on the response speed, but within the given test range, the pulse width was the main influencing factor. As the pulse width shortened, a slight decrease in the floating gate charge level was detected. However, for channel layer thicknesses of 20 nm, 40 nm, and 60 nm, the floating gate charge levels of UltraRAM varied minimally within a relatively long range of pulse widths, indicating that the influence of the channel layer thickness on the response speed might be relatively small.

It should be noted that when the pulse width was reduced to below 10 ns, the floating gate charge levels sharply decreased, and under pulse widths of 1 ns and 500 ps, the floating gate charge levels were close to the floating gate charge level in the idle state. This result means that UltraRAM cannot perform normal write and erase operations under such short pulse widths.

In conclusion, through our research and analysis, it can be inferred that the proposed improvement scheme of thinning the channel layer thickness has a positive impact on the performance of UltraRAM, including increasing the storage window and improving the device’s operating voltage. Furthermore, although the improvement scheme has some impact on the device’s response speed, it is minimal within a reasonable range of pulse widths. However, the current speed still falls short of the ideal speed and requires further research and optimization.

In addition to non-volatile and low-voltage write and erase operations, low switching energy is a critical memory feature and a major challenge for the competition between new memory technologies and DRAM and Flash. Similar to traditional memory, our devices are based on the principle of charge storage, so the switching energy depends on the energy required for the capacitor to charge and discharge: low-voltage switching means low-energy switching. In fact, since UltraRAM and Flash have similar structures; assuming the same gate size and capacitor conditions, the switching energy of UltraRAM is about 64 times lower than that of Flash and even lower than that of DRAM (for the same device size) due to the fact that the switching energy is proportional to the product of the capacitance value and the square of the operating voltage (E = 0.5 × CV^2^).

According to theoretical evaluations, for a 10 µm × 10 µm (gate size) device, the CG-FG capacitance is approximately 10^−12^ F, and the switching energy is about 2 × 10^−12^ J. As the device size decreases, this value will decrease significantly. For example, at a 20 nm process node, the switching energy is about 10^−17^ J, which is 100 times lower than DRAM and 1000 times lower than Flash. To the best of the authors’ knowledge, this unique memory technology demonstrates unparalleled potential for ultra-low switching energy.

Table 1 presents a performance comparison between UltraRAM and three other non-volatile memories. It can be observed that UltraRAM exhibits more significant advantages in terms of operating voltage and switching energy. Additionally, UltraRAM maintains a relatively good level of non-volatility while consuming less energy thanks to the quantum well tunneling mechanism. Moreover, UltraRAM theoretically possesses an extremely fast response speed, surpassing FeRAM and ReRAM by at least an order of magnitude. These advantages not only indicate the significant research potential and development prospects of UltraRAM but also suggest its potential to play a crucial role in the Internet of Things (IoT) domain.

## 4. Conclusions

This work provides an in-depth analysis of the structure, storage mechanism, and optimization scheme of UltraRAM, leading to the following conclusions. First, the advantages of UltraRAM, including a larger storage window and lower operating voltage, were validated, supported by the verification of the band structure and storage principles. However, issues of storage window clarity and speed limit the development of UltraRAM in large-scale array applications. To effectively address the problem of a small storage window, an improvement scheme was proposed, namely, thinning the channel layer thickness to increase the storage window. Three sets of channel layer thicknesses were examined, and the feasibility of the improvement scheme was validated by carrying out simulation-based experiments. The results demonstrated a significant 50% increase in the storage window after thinning the channel layer, representing a notable improvement. Additionally, data storage under the application of a 10 ns pulse width was tested, demonstrating the ability of this scheme to achieve data storage operations with shorter pulse widths. In conclusion, this study’s improvement scheme successfully enlarged the storage window of UltraRAM and achieved data storage with a 10 ns pulse width. Our work provides valuable pieces of evidence and a foundation for the further development of UltraRAM. However, further research and optimization are still undoubtedly needed to enhance the speed performance of the memory device and explore other improvement schemes to meet the requirements of large-scale array applications.

## Figures and Tables

**Table 1:** Performance comparison.

	3D NAND-Flash	FeRAM	ReRAM	UltraRAM
Cell elements	1T	1T1C	1T1R	1T
Voltage/V	≥10	≤3	≤3	≤2.6
Switching energy/J	10^−14^	10^−11^	10^−11^	10^−17^
Switching time	≥10 μs	50 ns	10–100 ns	≤10 ns
Endurance	10^5^	10^12^	10^6^–10^12^	>10^7^

## Data Availability

Data are contained within the article.
